# Complete Genome Sequence of Foot-and-Mouth Disease Virus Serotype O Isolated from Bangladesh

**DOI:** 10.1128/genomeA.01253-13

**Published:** 2014-02-06

**Authors:** Munawar Sultana, Mohammad Anwar Siddique, Samina Momtaz, Arafat Rahman, Huzzat Ullah, Shuvro Prokash Nandi, M. Anwar Hossain

**Affiliations:** Department of Microbiology, University of Dhaka, Dhaka, Bangladesh

## Abstract

Foot-and-mouth disease (FMD) is a highly infectious enzootic disease caused by FMD virus. The complete genome sequence of a circulatory FMD virus (FMDV) serotype O isolated from Natore, Bangladesh, is reported here. Genomic analysis revealed antigenic heterogeneity within the VP1 region, a fragment deletion, and insertions at the 5′ untranslated region (UTR) and 3A region compared to the genome of the available vaccine strain.

## GENOME ANNOUNCEMENT

Foot-and-mouth disease (FMD) is a severe plague in animal farms affecting domestic and wild cloven-hoofed animal species (1, 2). The etiological agent, FMD virus (FMDV), is included in the *Picornaviridae* family, genus *Apthovirus*, and it exists in seven immunologically distinct serotypes (Euroasiatic serotypes A, O, and C, Asia 1, and South African territories [SAT] 1 to 3), with multiple subtypes (3). FMDV type O is the pandemic serotype that is grouped into eight topotypes, i.e., Cathay, Middle East-South Asia (ME-SA), South-East Asia (SEA), Europe-South America (Euro-SA), Indonesia-1 and -2 (ISA-1 and -2), East Africa (EA), and West Africa (WA), based on 15% nucleotide differences (4). In Bangladesh, the serotype O Ind 2001 sublineage of the ME-SA topotype viruses was detected to be homogenously distributed across the regions during the recent outbreak in 2012 (5).

Here, we report a genomic study of the recent representative of the circulatory FMDV serotype O strain (Bangladesh/Natore/Haripur-156/2013 [BAN/NA/Ha-156/2013]) that was isolated on 6 July 2013 from an oral blister of an infected cow from Natore, Bangladesh, during an outbreak. Viral RNA was extracted from the cell culture supernatant using the BHK-21 cell line, and cDNA was synthesized with random and oligo(dT) primers. Amplifications were done using internal primer pairs to generate 16 overlapping amplicons spanning the entire viral genome. The sequencing reactions were analyzed in an ABI genetic analyzer, and the contigs were assembled using Seqman version 7.0 (DNAStar Lasergene, USA). Phylogenetic analysis was done using the MEGA 5.2 software.

The complete genome of strain BAN/NA/Ha-156/2013 is 8,131 nucleotides (nt) in length, including a 1,020-nt 5′ untranslated region (5′ UTR) with a 15-nt poly(I:C) tract, a 6,999-nt open reading frame (ORF), and a 112-nt 3′ UTR with a ≥21-nt poly(A) tail. The homologous comparison and phylogenetic analysis of the nucleotide and deduced amino acid (aa) sequences between strain BAN/NA/Ha-156/2013 and other FMDV strains available in the database revealed that the strain is clustered within the IND 2001 linage of the ME-SA topotype of FMDV serotype O. Strain BAN/NA/Ha-156/2013 shares 88% nucleotide homology (in relation to the VP region) with the currently available vaccine strain (FMDV O strain India/R2/75; GenBank accession no. AF204276.1). Compared to the vaccine serotype, critical amino acid substitutions were determined at the VP1 GH loop (positions 136–150): D138E, S140A, and I144V, which are responsible for antigenic heterogeneity. Moreover, an insertion of 11 amino acid residues in the 3A segment and the deletion of a total of 81 base stretches at the S-fragment along with a 42-nt insertion within the 5′ UTR were found in comparison to the NCBI FMDV type O RefSeq sequence (accession no. NP_658990.1).

The FMDV serotype O isolate BAN/NA/Ha-156/2013 is a local strain that is a potential vaccine candidate, belonging to the ME-SA topotype. We present here the first whole-genome sequence data for a local FMDV strain of serotype O from Bangladesh. The complete genetic information will be helpful for switching the available vaccine candidate, although its immunogenicity and antigenicity need to be assessed further.

### Nucleotide sequence accession number.

The complete genome sequence of FMDV isolate BAN/NA/Ha-156/2013 has been deposited in GenBank under the accession no. KF985189.
